# Genomic Analysis of Non-B Nucleic Acids Structures in SARS-CoV-2: Potential Key Roles for These Structures in Mutability, Translation, and Replication?

**DOI:** 10.3390/genes14010157

**Published:** 2023-01-06

**Authors:** Stefan Bidula, Václav Brázda

**Affiliations:** 1 School of Pharmacy, University of East Anglia, Norwich Research Park, Norwich NR4 7TJ, UK; 2 Institute of Biophysics of the Czech Academy of Sciences, 61265 Brno, Czech Republic

**Keywords:** SARS-CoV-2, inverted repeats, G-quadruplex, pseudoknot, spike protein, mutation, adaptation

## Abstract

Non-B nucleic acids structures have arisen as key contributors to genetic variation in SARS-CoV-2. Herein, we investigated the presence of defining spike protein mutations falling within inverted repeats (IRs) for 18 SARS-CoV-2 variants, discussed the potential roles of G-quadruplexes (G4s) in SARS-CoV-2 biology, and identified potential pseudoknots within the SARS-CoV-2 genome. Surprisingly, there was a large variation in the number of defining spike protein mutations arising within IRs between variants and these were more likely to occur in the stem region of the predicted hairpin stem-loop secondary structure. Notably, mutations implicated in ACE2 binding and propagation (e.g., ΔH69/V70, N501Y, and D614G) were likely to occur within IRs, whilst mutations involved in antibody neutralization and reduced vaccine efficacy (e.g., T19R, ΔE156, ΔF157, R158G, and G446S) were rarely found within IRs. We also predicted that RNA pseudoknots could predominantly be found within, or next to, 29 mutations found in the SARS-CoV-2 spike protein. Finally, the Omicron variants BA.2, BA.4, BA.5, BA.2.12.1, and BA.2.75 appear to have lost two of the predicted G4-forming sequences found in other variants. These were found in *nsp2* and the sequence complementary to the conserved stem-loop II-like motif (S2M) in the 3′ untranslated region (UTR). Taken together, non-B nucleic acids structures likely play an integral role in SARS-CoV-2 evolution and genetic diversity.

## 1. Introduction

When we consider the structure of nucleic acids, our first thoughts are of the iconic DNA beta-helical structure. However, nucleic acids are structurally diverse and can be found in a wide range of topologies and conformations within both living and non-living entities. Non-B nucleic acids have been identified as important regulators in fundamental biological processes and have emerged as novel therapeutic targets within infection and disease.

There is growing evidence that these non-canonical nucleic acids structures, such as G-quadruplexes (G4s), cruciforms, hairpins, and pseudoknots, may contribute to both the functional biology and mutational variability of humans, animals, plants, and microorganisms [1,2,3,4,5]. Inverted repeats (IRs) constitute a sequence of nucleotides followed downstream by its reverse completed sequence, often separated by a ‘loop’ sequence. Viral origins of replication and bacterial plasmids are found to be enriched with IRs [6,7]. These IR sequences can fold into a hairpin stem-loop structure or palindrome in single-stranded nucleic acids. This can significantly contribute to genomic instability and mutation [8]. Furthermore, they have been implicated in a wide range of biological processes, such as replication, transcription, and DNA repair [9,10]. IRs also regulate RNA processing in animals and plants, and transcripts containing IRs are processed to produce small RNAs which silence genes [11,12]. IRs are also an important component of pseudoknots: a common structural motif in RNA formed of two nested stem-loops [13]. Pseudoknots have been found to be present in viruses whereby they contribute to viral translation, replication, and can also induce frameshifts [5]. Pseudoknots have also been shown to act as binding sites for proteins and may act as regulatory switches in response to environmental signals. They are highly conserved amongst viruses, and, as such, they are beginning to emerge as a potential antiviral target for SARS-CoV-2 [14].

G4s are four-stranded nucleic acids structures that arise in guanine-rich regions of RNA/DNA, and are formed in sequences composed of four runs of ≥two guanines separated by a nucleotide loop (e.g., **GG**AT**GG**AT**GG**AT**GG**) [15]. Here, four guanines associate via Hoogsteen hydrogen bonding to form a G-tetrad. These G-tetrads stack upon one another and are stabilised by a metal cation (e.g., K^+^) to form the G4 secondary structure. These structures have been gaining interest recently as antimicrobial targets, due to their demonstrable roles in the regulation of fundamental biological processes such as transcription, translation, replication, and alternative gene splicing [15]. Indeed, G4s have arisen as promising drug targets within bacteria, viruses, parasites, and fungi [16,17,18,19]. 

Goswami and colleagues recently highlighted that SARS-CoV-2 hot-spot mutations were significantly enriched within IRs in the Wuhan reference genome, and hypothesised that IRs could contribute to further mutational drive [20]. This hypothesis was confirmed in additional variants, but in-depth analyses of IRs in more recently identified variants have not been conducted [21]. Moreover, G4s have recently arisen as promising targets to treat SARS-CoV-2 infections [22]. The important roles of these non-canonical nucleic acids structures in SARS-CoV-2 are only just starting to become apparent. Thus, critical biological insights into the roles these structures may have in SARS-CoV-2 could help with understanding the biology of this virus and unveil novel druggable targets to treat these infections. In this article, we analyse SARS-CoV-2 genomes for the presence of IRs, pseudoknots, and G4s, with the aim of stimulating new schools of thought and identifying future experimental directions for the fields of nucleic acids biology and virology. 

## 2. Materials and Methods

### 2.1. Selection of Sequences

Representative genomes for the currently circulating variant of concern (Omicron), formerly circulating variants of concern (Alpha, Beta, Gamma, and Delta), 9 formerly monitored variants (Epsilon, 20A, Kappa, Iota, 20B, Eta, Theta, Lambda, and Mu), and the Wuhan reference strain were analysed. The FASTA sequences for the entire genomes and the *S* genes encoding the SARS-CoV-2 spike glycoproteins for each were obtained from the National Center for Biotechnology Information (NCBI; last accessed 19 December 2022). Only complete genomes and sequences were used for analysis. Representative genomes were used as there was negligible variation between the locations of predicted non-B structures amongst all genomes from the same variant. The accession numbers of the genomes analysed can be found in Table S1 and the genome information can be found in the Supplementary Materials.

### 2.2. Detection of Mutations within IRs, Prediction of Pseudoknot Formation, and G4-Analysis

To quantify the number of predicted IRs within the *S* genes, the FASTA sequences were analysed using the Palindrome Analyser web server (http://palindromes.ibp.cz/#/en/index; (last accessed on 19 December 2022) [23]) using the default settings (size: 6–30 bp, spacer: 0–10 bp, and mismatches: 0, 1). Defining, shared, and unique mutations were identified via CoVariants (https://covariants.org/; (last accessed on 19 December 2022) [24]), which collates raw data provided by the Global Initiative on Sharing All Influenza Data ((GISAID); [25]). Prior to post-analysis, FASTA sequences of the variants’ *S* genes were aligned to the Wuhan reference sequence using Clustal Omega (EMBL-EBI) to account for any effects of the deletion mutations and differences in nucleotide number. Mutations were noted to have occurred within an IR only if the mutation site fell within the stem or loop region of the predicted IR. Pseudoknot formation was predicted using ProbKnot within the RNAstructure program as described previously [26,27]. Pseudoknot predictions were performed using 1 iteration and a minimum helix length of 3. The ProbKnot CT files containing the predicted pseudoknot structures are provided in the Supplementary Materials. The presence of G4-forming sequences in the SARS-CoV-2 genomes was determined via QGRS Mapper using the search options of max length = 30, minimum group size = 2, and loop size = 0–12 [28]. 

### 2.3. Statistical Analysis

Data comparing groups were first tested for normality via a Shapiro–Wilk normality test prior to analysis via either an unpaired Student’s *t*-test or one-way ANOVA depending upon the number of variables. Significance was given as any value < 0.05. 

## 3. Results

### 3.1. There Is a Large Variation in the Number of Defining Mutations Falling within IRs between SARS-CoV-2 Variants

In-depth analyses of IRs in more recently identified SARS-CoV-2 variants have not been conducted [21]. Therefore, we first identified the presence of IRs in the entire genome and *S* genes of the currently circulating variant of concern (Omicron), formerly circulating variants of concern (Alpha, Beta, Gamma, and Delta), and nine formerly monitored variants (Epsilon, 20A, Kappa, Iota, 20B, Eta, Theta, Lambda, and Mu) to offer insight into whether SARS-CoV-2 was continuing to mutate as expected.

**Figure 1:**
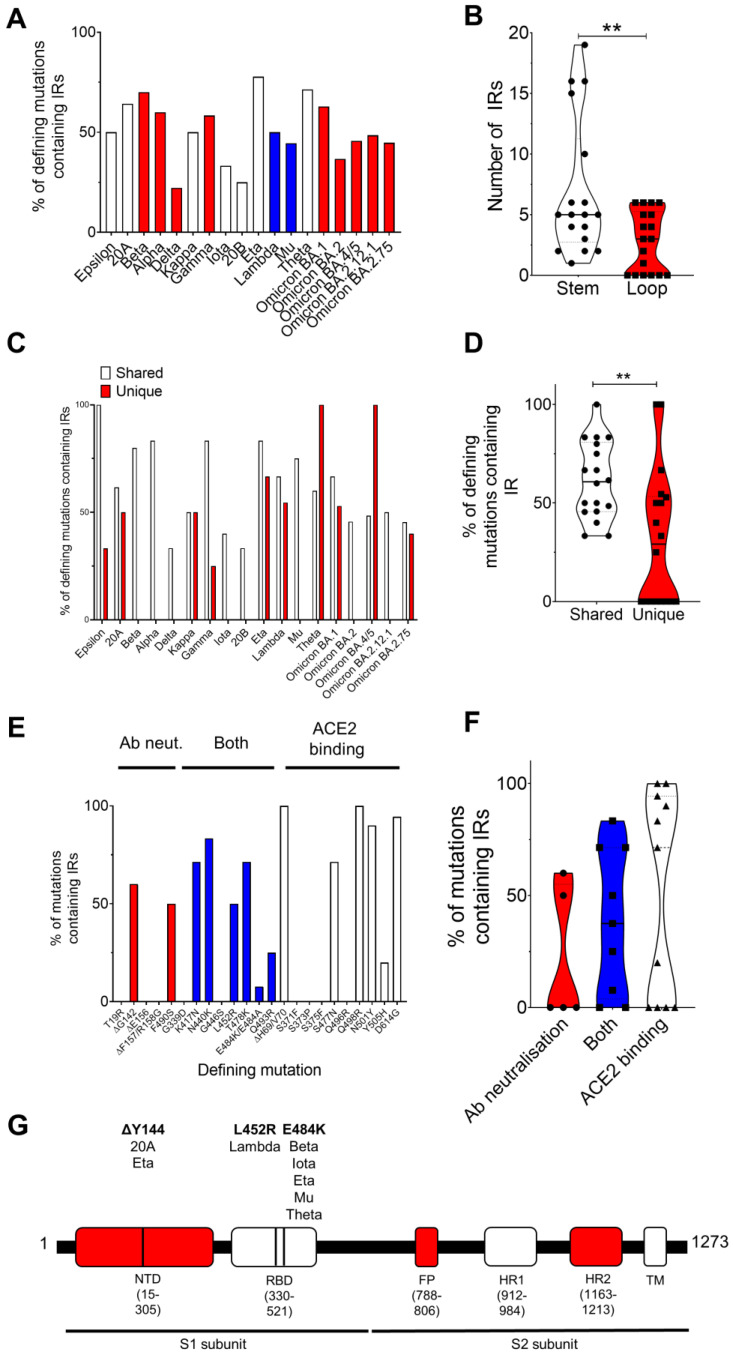
Defining SARS-CoV-2 spike protein mutations involved in ACE2 binding and antibody neutralization are found within inverted repeats (IRs) and predicted pseudoknots. (**A**) The percentage of defining spike protein mutations falling within IRs differed considerably between the variants investigated. (**B**) Spike protein mutations were more likely to fall within the stem, rather than the loop, of the predicted secondary hairpin stem-loop structure. (**C**,**D**) Spike protein mutations which frequently occurred in more than one variant (shared) were significantly more likely to occur within IRs compared to mutations which were unique to a variant. (**E**,**F**) Spike protein mutations associated with ACE2 binding were more frequently found within IRs, whereas mutations associated with antibody neutralization were found infrequently within IRs. Mutations implicated in both are highlighted in blue. (**G**) In the Wuhan strain, pseudoknots were predicted to form within, or within 20 bp, of the ΔY144, L452K, and E484K mutations involved in antibody neutralization and ACE2 binding within the NTD and RBD of the SARS-CoV-2 spike protein. Those variants sharing these predicted pseudoknots are listed. IRs were detected using Palindrome Analyser with the default settings (size: 6–30 bp, spacer: 0–10 bp, and mismatches: 0, 1) and pseudoknot predictions were performed using ProbKnot in the RNAstructure program. ** indicates a significant difference (*p* < 0.05). Data were tested for normality via Shapiro–Wilk prior to comparison via an unpaired Student’s *t*-test. NTD, N-terminal domain; RBD, receptor-binding domain; FP, fusion peptide; HR, heptapeptide repeat sequence; TM, transmembrane domain.

We found no significant difference in the number of IRs in the complete genome or in the *S* genes between variants (Table S1; Figure S1). Unexpectedly, we did find that the number of defining spike mutations occurring within IRs was largely varied between variants (Table 1; Figure 1A). Defining spike mutations of the Delta (22.2%), 20B (25%), and Iota (33.3%) variants were least likely to be found within IRs, but spike mutations of the Beta (70%), Eta (77.7%), and Theta (71.4%) variants were frequently located within IRs (Table 1; Figure 1A). Regarding specific mutations, the D614, N501, ΔY144, ΔG142, T478, N440, K417, Q498, and ΔH69/V70 mutations were most frequently found within IRs (Table 1). Interestingly, we also found that defining mutations shared by the variants (e.g., ΔD69/V70, ΔY144, N501Y, and D614G) were significantly more likely to be found within IRs compared to those unique to a variant, such as A570D, T716I, and S982A in the Alpha variant and ΔE156/F157 and R158G in the Delta variant (Figure 1C,D). We also observed a preference for the defining mutation to be found within the stem rather than the loop of the IR (Figure 1B; Table S1). Thus, it appeared that IRs play an integral role in driving the mutational diversity of spike protein mutations amongst variants. However, why some mutations were preferentially found within IRs and not others was unknown.

Many of the mutations under investigation have now been implicated in ACE2 binding, antibody neutralization, or both [29,30,31,32,33,34]. We observed that defining mutations contributing to ACE2 binding, such as ΔH69/V70, N501Y, and D614G, were regularly found to occur within IRs (100.0%, 90.0%, and 94.4% of instances where this mutation was present were found within IRs, respectively). Conversely, mutations significantly contributing to antibody neutralization, such as T19R, ΔE156, ΔF157, R158G, and G446S, were not found within IRs (Figure 1E,F; Table S2). 

### 3.2. Pseudoknots Are Predicted to Occur near the Sites of Several Key Mutations

In the Wuhan reference strain, pseudoknot prediction algorithms determined the presence of potential pseudoknots within the sites where the A27S, E484K, and S704L mutations occur (Figure 1G). Moreover, pseudoknots were predicted to form within 30 bp of the sites where T20N, ΔL24-P26, Q52R, G75V, T76I, D80A, R158G, R190S, G257S, R346K, S371L, S373P, S375F, T376A, K417N, G446S, L452R, S477N, T478K, F486V, F490S, A570D, D614G, Q677H, A701V, D796Y, F888L, D950N, and Q954H arise in other variants (Supplementary genome information. 

### 3.3. G4 Are Predicted to Form on the Negative Strand Genome in SARS-CoV-2 

**Figure 2:**
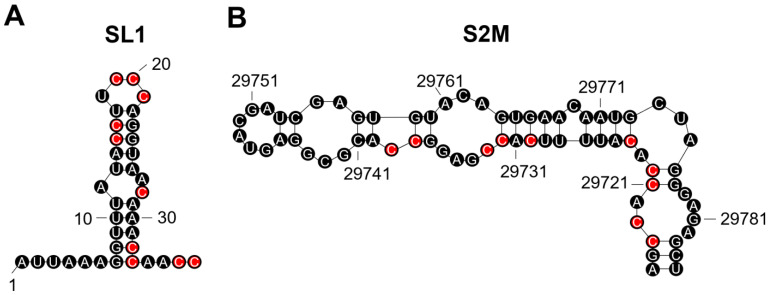
The stem-loop 1 (SL1) and stem-loop II-like motif (S2M) regions in the SARS-CoV-2 untranslated regions (UTRs) contain sequences on their positive strand which may allow G-quadruplex (G4) formation on the negative strand. Both the SL1 (**A**) and S2M (**B**) regions contain C-tracts on their positive sense RNA strand which may allow the formation of a G4 to occur on the negative sense strand. The cytosines which may be involved are highlighted in red. The reverse complemented sequence on the negative strand would be GGAAGGGUCCAUUGUUUGGUUGG (SL1) and GGUGGUGTAAAAGUGGCUCCGG (S2M). Both sequences have the potential to form G4s and G4-formation on the negative strand may prevent viral translation and replication.

We analysed SARS-CoV-2 genomes from all variants and identified putative G4-forming sequences in the *nsp1*, *nsp2*, *nsp3*, *nsp4*, *nsp10*, *nsp12*, *nsp14*, *nsp15*, *nsp16*, *S*, and *N* genes and both the 3′ and 5′ untranslated regions (UTRs; Table 2 and Table S3). Notably, the predicted conserved G4-forming sequence (**GG**AA**GGG**UCCAUUGUUU**GG**UU**GG**; reverse complemented sequence) falls within stem-loop 1 region (SL1; Table 2; Figure 2A). The G4 in the 3′UTR is predicted to fall with the stem-loop II-like motif (S2M), a motif conserved amongst positive single-stranded RNA viruses from the *Astroviridae*, *Calciviridae*, *Picornaviridae*, and *Coronaviridae* (Table 2; Figure 2B) [35]. Both the predicted UTR sequences are the reverse complement and the G4 would be found on the negative sense strand. Interestingly, the Omicron variants BA.2, BA.4, BA.5, BA.2.12.1, and BA.2.75 appear to have lost two of the predicted G4-forming sequences in other variants. These were found in *nsp2* and the 3′UTR S2M motif. 

## 4. Discussion

We found that there was significant diversity in the percentage of defining spike protein mutations occurring within IRs between variants. Mutations linked to infectivity were more likely to arise within IRs compared to those associated with antibody neutralization. Moreover, pseudoknots were predicted to form close to key spike protein mutations and G4s were predicted to form within two conserved regions within the 3′ and 5′ UTRs. 

The SARS-CoV-2 mutations found most likely to occur within IRs amongst all variants were the ΔH69/V70, N440K, N501Y, and D614G mutations; all of which have been implicated in increased fitness and infectivity [36,37,38,39,40]. Mutations implicated in ACE2 binding and propagation were found to frequently occur within IRs, whilst mutations involved in antibody neutralization and reduced vaccine efficacy were rarely found within IRs. Although not significant, these data suggest that mutations linked to antibody neutralization may occur more frequently outside of IRs and are probably evoked due to the external pressures of vaccines and antibodies, rather than spontaneous mutation. 

Surprisingly, the D614G mutation was found within an IR for all variants except for Omicron BA.2, BA.4, and BA.5. The D614G mutation has been shown to enhance infectivity but it has also been shown to enhance susceptibility to vaccines and antibody neutralization. Notably, the Omicron BA.2, BA.4, and BA.5 variants also display increased resistance to neutralizing antibodies [41,42,43,44]. However, this is unlikely to be due to the loss of this IR sequence and more likely to be due to the involvement of the S371F, D405N, R408S, F486V, and L452R mutations. However, of these mutations, only the F486V and L452R mutations were found within IRs, further supporting our claim that antibody neutralizing mutations occur with less frequency within IRs. 

In the Wuhan reference strain, pseudoknot prediction algorithms determined the presence of potential pseudoknots within the sites where the A27S, E484K, and S704L mutations occur and in 29 additional mutations amongst the variants tested. The E484 mutation is particularly noteworthy as this mutation has been shown to arise with high frequency in the presence of antibodies [45]. However, whether the external influence exerted by antibodies can induce pseudoknot formation is unknown. It is well known that the pseudoknot in the ORF1 polyprotein of SARS-CoV-2 can induce frameshifts, whilst the conserved pseudoknot in the coronavirus 3′UTR is involved in viral replication [46]. These are two such key examples, but one can hypothesise that these examples are the tip of the iceberg, and that pseudoknots have important roles throughout the entire viral genome. Future studies could investigate whether antibody binding acts as an environmental trigger for pseudoknot formation/prevention and whether this influences further mutational drive. 

It was previously demonstrated that the SARS-CoV-2 genome contained fewer G4s than the SARS-CoV genome and this has been suggested to be energetically favourable, as G4s can represent a barrier to translation and replication [47,48]. Moreover, the frequency of G4s in a viral genome is associated with whether infection is chronic or acute [28]. The G4s in the *nsp1*, *nsp3*, *nsp10*, *S*, and *N* genes have previously been shown to form in vitro [48]. However, the roles these G4s might play in controlling the biological functions of these genes have not been fully addressed. Of particular interest are the G4-forming sequences found in the UTRs of SARS-CoV-2. Both predicted G4 sequences would be found on the negative strand. It has recently been identified that SARS-CoV-2 negative strands have protein-coding potential, and they are known to be involved in replication [49]. Thus, the negative strand may be targeted by G4-stabilising compounds to prevent translation of proteins on the negative sense strand and subsequent SARS-CoV-2 replication cycles. Indeed, several G4-stabilisers have been found to bind to SARS-CoV-2 RNA and G4-stabilising compounds have recently been demonstrated to be antiviral in mouse models of infection [50,51,52], highlighting the therapeutic potential of targeting G4s in SARS-CoV-2 infections.

It has recently been shown that the conserved SL1 region in the 5′UTR of SARS-CoV-2 represents a potential drug target [53]. The authors demonstrated that a locked nucleic acid (LNA) antisense oligonucleotide to the SL1 region could inhibit viral translation, prevent lethality in mice expressing ACE2, and make SARS-CoV-2 vulnerable to non-structural protein 1 (Nsp1) translation suppression [53]. Chowdhury et al., recently demonstrated that LNA probes can promote disruption of the secondary G4 structure [54]. Therefore, it is likely that the LNA oligonucleotide used against the SL1 region could also disrupt the G4 predicted to form on the negative strand. This suggests that this conserved G4-forming sequence could be important in promoting viral translation and molecules designed to disrupt this G4 might have therapeutic potential. 

The S2M region has previously been described as a recombination hotspot in SARS-CoV-2 compared with other positive single-stranded RNA viruses [55]. It is well-established that G4s can contribute to genome instability, and it is likely that this G4-forming sequence in past variants has contributed to the genetic variability observed within the S2M region of the new variants. On another note, the presence of two potential TAGGGA microsatellites in close vicinity to this region probably also contributes to the genetic variability within this region due to their high mutation rates. Finally, interferon-β (IFN-β) can inhibit SARS-CoV-2 replication and Nsp2 has recently been shown to repress the translation of IFN-β [56]. The presence of G4-forming sequences in an mRNA can prevent translation, and the loss of the predicted sequences in the recent Omicron variants could provide some explanation for the increased replication of these variants. Thus, loss of the G4-forming sequence from *nsp2* might enhance the translation of Nsp2 and promote replication. 

## 5. Conclusions

Taken together, non-B nucleic acids structures are prevalent throughout the SARS-CoV-2 genome where they may play integral roles in promoting mutational diversity. Furthermore, it could be interesting to explore whether environmental pressures, such as the immune response and antibodies, influence the formation of IRs, G4s, and pseudoknots. Finally, targeting non-B nucleic acids structures in SARS-CoV-2 may disrupt viral biological processes and have therapeutic potential, although a much greater understanding of their biological roles in SARS-CoV-2 is required.

## Figures and Tables

**Table 1:** SARS-CoV-2 variants studied and defining spike mutations containing inverted repeats (IRs).

Variant	Pango Lineage	Defining Spike Protein Mutations	Defining Mutations within IRs
Epsilon	B.1.427	S13I W152C L452R D614G	W152C D614G
20A	B.1.620	ΔP26, ΔH69/V70, V126A, ΔY144, ΔL241-A243, H245Y, S477N, E484K, D614G, P681H, T1027I, D1118H	ΔH69/V70, V126A, ΔY144, ΔL241-A243, D614G, D1118H
Beta	B.1.351	D80A, D215G, ΔL241-A243, K417N, E484K, N501Y, D614G, A701V	ΔL241-A243, K417N, N501Y, D614G, A701V
Alpha	B.1.1.7	ΔH69/V70, ΔY144, N501Y, A570D, D614G, P681H, T716I, S982A, D1118H	ΔH69/V70, ΔY144, N501Y, D614G, D1118H
Delta	B.1.617.2	T19R, ΔE156/F157, R158G, L452R, T478K, D614G, P681R, D950N	D614G, D950N
Kappa	B.1.617.1	E154K, L452R, E484Q, D614G, P681R, Q1071H	E484Q, D614G, Q1071H
Gamma	P.1	L18F, T20N, P26S, D138Y, R190S, K417T, E484K, N501Y, D614G, H655Y, T1027I, V1176F	L18F, T20N, R190S, K417T, N501Y, D614G, V1176F
Iota	B.1.526	L5F, T95I, D253G, E484K, D614G, A701V	D614G, A701V
20B	B.1.1.519	T478K, D614G, P681R, T732A	D614G
Eta	B.1.525	Q52R, A67V, ΔH69/V70, ΔY144, E484K, D614G, Q677H, F888L	Q52R, A67V, ΔH69/V70, ΔY144, D614G, F888L
Lambda	C.37	G75V, T76I, ΔR246-G252, D253N, L452Q, F490S, D614G, T859N	G75V, T76I, ΔR246-G252, L452Q, F490S, D614G
Mu	B.1.621	T95I, Y144S, Y145N, R346K, E484K, N501Y, D614G, P681R, D950N	Y144S, Y145N, D614G, D950N
Theta	P.3	E484K, N501Y, D614G, P681R, E1092K, H1101Y, V1176F	N501Y, D614G, E1092K, H1101Y, V1176F
Omicron	BA.1	A67V, ΔH69/V70, T95I, ΔG142-Y144, Y145D, ΔN211, L212I, G339D, S371L, S373P, K417N, N440K, G446S, S477N, T478K, E484A, Q493R, Q496R, Q498R, N501Y, Y505H, T547K, D614G, H655Y, N679K, P681H, N764K, D796Y, N856K, Q954H, N969K, L981F	A67V, ΔH69/V70, ΔG142-Y144, Y145D, ΔN211, L212I, N440K, S477N, T478K, Q498R, N501Y, Y505H, D614G, N679K, N764K, D796Y, Q954H, N969K, L981F
Omicron	BA.2	T19I, ΔL24-P26, A27S, G142D, V213G, G339D, S371F, S373P, S375F, T376A, D405N, R408S, K417N, N440K, S477N, T478K, E484A, Q493R, Q498R, N501Y, Y505H, D614G, H655Y, N679K, P681H, N764K, D796Y, Q954H, N969K	G142D, V213G, N440K, S477N, T478K, Q498R, N501Y, N764K, D796Y, Q954H, N969K
Omicron	BA.4/BA.5	T19I, ΔL24-P26, A27S, ΔH69/V70, G142D, V213G, G339D, S371F, S373P, S375F, T376A, D405N, R408S, K417N, N440K, L452R, S477N, T478K, E484A, F486V, Q498R, N501Y, Y505H, D614G, H665Y, N679K, P681H, N764K, D796Y, Q954H, N969K	ΔH69/V70, G142D, V213G, N440K, L452R, S477N, T478K, F486V, Q498R, N501Y, H665Y, N764K, D796Y, Q954H, N969K
Omicron	BA.2.12.1	T19I, ΔL24-P26, A27S, G142D, V213G, G339D, S371F, S373P, S375F, T376A, D405N, R408S, K417N, N440K, L452Q, S477N, T478K, E484A, Q493A, Q498R, N501Y, Y505H, D614G, H655Y, N679K, P681H, S704L, N764K, D796Y, Q954H, N969K	G142D, V213G, D405N, K417N, N440K, L452Q, S477N, T478K, Q498R, N501Y, D614G, N679K, N764K, D796Y, Q954H, N969K
Omicron	BA.2.75	T19I, ΔL24-P26, A27S, G142D, K147E, W152R, F157L, I210V, V213G, G257S, G339H, S371F, S373P, S375F, T376A, D405N, R408S, K417N, N440K, G446S, N460K, S477N, T478K, E484A, R493Q, Q498R, N501Y, Y505H, D614G, H655Y, N679K, P681H, N764K, D796Y, Q954H, N969K	K147E, V213G, D405N, K417N, N440K, N460K, S477N, T478K, R493Q, Q498R, N501Y, D614G, N679K, N764K, D796Y, Q954H, N969K

**Table 2:** SARS-CoV-2 genes containing predicted G-quadruplex (G4)-forming sequences.

Gene Name/Region	Highest Scoring Sequence	+ or − Strand
*nsp1*	**GG**CTTT**GG**A**G**ACTCC**G**T**GG**A**GG**A**GG**	+
*nsp2*	**GG**T**G**TT**G**TT**GG**A**G**AA**GG**TTCC**G**AA**GG**	+
*nsp3*	**GG**ATAT**GG**TT**GG**TTT**GG**	−
*nsp4*	**GG**T**G**ATA**G**A**GG**TTT**G**T**GG**T**GG**TT**GG**	−
*nsp10*	**GG**TAT**G**T**GG**AAA**GG**TTAT**GG**	+
*nsp12*	**GG**AACCACTAAATTTTAT**GG**T**GG**TT**GG**	+
*nsp14*	**GG**TT**GGG**TT**GG**TTTT**G**AT**G**TT**G**AA**GG**	+
*nsp15*	**GG**A**G**CCCACAA**GG**TAATCCA**GG**T**GG**	+
*nsp16*	**GG**A**G**AAATA**G**TACAACAT**GG**AAT**GG**C**GG**	+
*S*	**GG**CTTATA**GG**TTTAAT**GG**TATT**GG**	+
*N*	**GG**CT**GG**CAAT**GG**C**GG**	+
3′UTR	**GG**U**GG**U**G**TAAAA**G**U**GG**CUCC**GG**	−
5′UTR	**GG**AA**GGG**UCCAUU**G**UUU**GG**UU**GG**	−

## Data Availability

Data supporting the study are available upon request.

## Supplementary Materials

The following supporting information can be downloaded at: https://www.mdpi.com/article/10.3390/genes14010157/s1, Figure S1: The total number of IRs in the whole genome and spike protein; Table S1: SARS-CoV-2 spike protein mutations within inverted repeats (IRs); Table S2: Mutations involved in ACE2 binding and antibody neutralization found within IRs; and Table S3: Putative G4-forming sequences in SARS-CoV-2; Supplementary genome information.
