# Supplementary figures and images for: Genomic Analysis of Non-B Nucleic Acids Structures in SARS-CoV-2: Potential Key Roles for These Structures in Mutability, Translation, and Replication?
Source: Genes (Basel). 2023 Jan 6;14(1):157. doi: 10.3390/genes14010157 (PMC9859294; doi:10.3390/genes14010157)

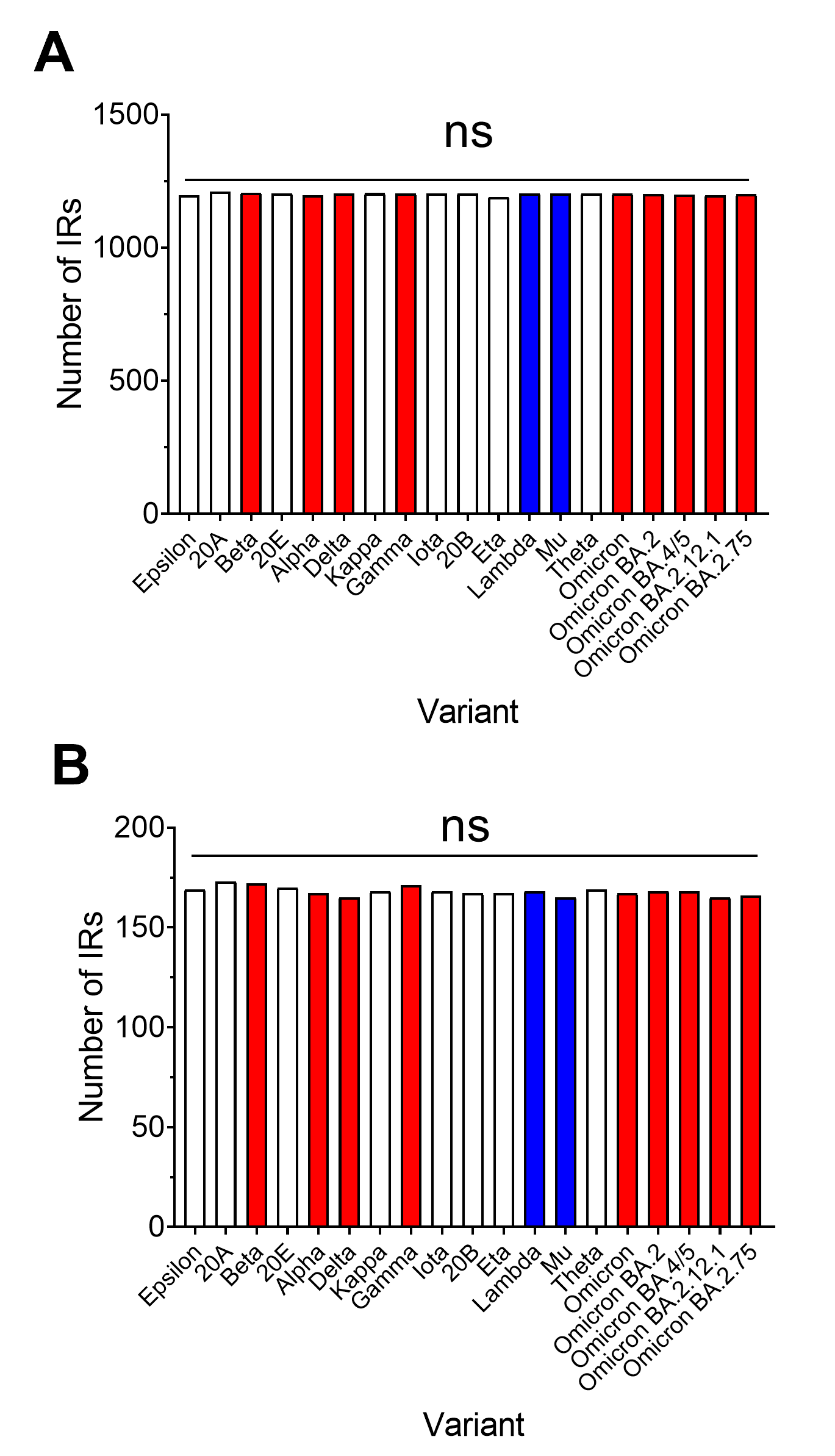

Supplement: Supplementary file 1 [file genes-14-00157-s001.zip › Figure_S1.tif]
